# Glycolipids from seaweeds and their potential biotechnological applications

**DOI:** 10.3389/fcimb.2014.00174

**Published:** 2014-12-17

**Authors:** Erwan Plouguerné, Bernardo A. P. da Gama, Renato C. Pereira, Eliana Barreto-Bergter

**Affiliations:** ^1^Laboratório de Produtos Naturais e Ecologia Química Marinha, Departamento de Biologia Marinha, Instituto de Biologia, Universidade Federal FluminenseNiterói, Brazil; ^2^Laboratório de Química Biológica de Microrganismos, Departamento de Microbiologia Geral, Instituto de Microbiologia, Universidade Federal do Rio de JaneiroRio de Janeiro, Brazil

**Keywords:** seaweeds, glycolipids, MGDG, DGDG, SQDG, biological activity, marine macroalgae

## Abstract

Marine macroalgae, or seaweeds, are a formidable source of natural compounds with diverse biological activities. In the last five decades it has been estimated that more than 3000 natural compounds were discovered from these organisms. The great majority of the published works have focused on terpenoids. In comparison, glycolipids are a neglected class of macroalgal secondary metabolites therefore remaining as a largely unknown reservoir of molecular diversity. Nevertheless, the interest regarding these compounds has been growing fast in the last decades as activities of ecological or pharmaceutical interest have been highlighted. This paper will review recent work regarding isolation and structural characterization of glycolipids from seaweeds and their prospective biological activities.

## Introduction

For the last five decades, both chemists and biologists have carried out an intense effort regarding marine natural products (Bhakuni and Rawat, 2005; Blunt et al., 2014). Such dedication has resulted in the discovery of more than 20,000 compounds from marine microorganisms, invertebrates, and macroalgae (Hu et al., 2011). Marine organisms appear then as a formidable source of natural products. While products of primary metabolism like amino acids, carbohydrates and proteins, are vital for maintaining life processes, others such as alkaloids, phenolics, steroids, terpenoids, are secondary metabolites that have ecological, toxicological, and pharmacological significance (Maschek and Baker, 2008). Bioactivities such as antiherbivory, antifoulant, antifungal, antitumor, antimicrobial, and antiparasitic effects have been highlighted for marine natural products (Noda et al., 1990; Deal et al., 2003; Bhadury and Wright, 2004; Cheung et al., 2014).

**Figure 1:**
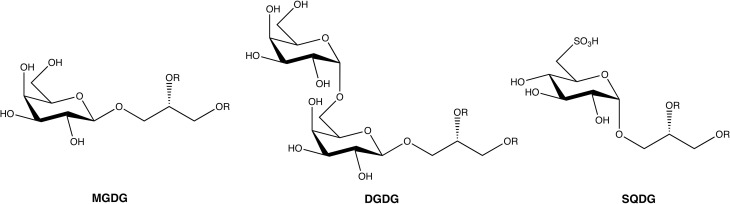
**General structure of the three main glycoglycerolipids from seaweeds**. R, acyl chain.

With an estimation of more than 30,000 species identified around the world, marine macroalgae, or seaweeds, constitute a huge source of natural compounds with diverse biological activities (Guiry, 2012). In the last 50 years it has been estimated that more than 3000 natural compounds were discovered from these organisms (Leal et al., 2013). The great majority of the published works have focused on terpenoids, phenolic compounds, or polysaccharides. Glycolipids represent a less studied class of metabolites with recently growing interest. Seaweeds biosynthesize three major types of glycolipids: monogalactosyldiacylglycerides (MGDGs), digalactosyldiacylglycerides (DGDGs), and sulfoquinovosyldiacylglycerides (SQDGs) (Figure 1).

These glycoglycerolipids are present in chloroplasts of eukaryotic algae where MGDGs and DGDGs are the most abundant lipids of the thylakoid membrane and appear to play a crucial role in photosynthesis (Hölzl and Dörmann, 2007).

This work will present a concise review of studies from the last 15 years regarding the isolation and structural characterization of bioactive glycolipids from marine macroalgae.

## Ochrophyta

The MGDG isolated from *Petalonia binghamiae* was characterized as a potent inhibitor of the activities of mammalian DNA polymerase α (Mizushina et al., 2001).

SQDG and DGDG from the Japanese macroalga *Sargassum horneri* were found to induce apoptosis of the human colon carcinoma Caco-2 cell when associate with sodium butyrate (Hossain et al., 2005).

The acetone fraction of *Sargassum thunbergii* extract exhibited moderate antifungal effects on *Candida albicans*. Further investigation of this extract led to the isolation of four MGDGs, two of them newly described as (2S)-1-*O*-(5Z,8Z,11Z,14Z,17Z-eicosapentaenoyl)-2-*O*-(9Z,12Z,15Z-octadecatrienoyl)-3-*O*-β-D-galactopyranosyl- sn-glycerol and (2S) -1-*O*- (9Z,12Z, 15Z-octadecatrienoyl)-2-*O*-(6Z,9Z,12Z,15Z-octadecatetraenoyl)-3-*O*-β-D-galactopyranosyl-sn-glycerol (Kim et al., 2007).

Two glucopyranosyldiacylglycerols were isolated from *Sargassum fulvellum*. The two compounds were identified to be 1-*O*-palmitoyl-2-*O*-oleoyl-3-*O*-(α-D-glucopyranosyl)-glycerol and 1-*O*-myristoyl-2-*O*-oleoyl-3-*O*-(α-D-glucopyranosyl)-glycerol and showed fibrinolytic activity in the reaction system of single chain urokinase-type plasminogen activator and plasminogen (Wu et al., 2009).

The dichloromethane-methanol (7/3) extract of *Lobophora variegata* from the Yucatan coast (Mexico) demonstrated activity against the protozoa *Trichomonas vaginalis*, with an IC_50_ value of 3.2 μg/ml (Cantillo-Ciau et al., 2010). Further fractionation of that extract was undergone and led to a chloroform fraction that showed activity against the protozoa *T. vaginalis*, *Giardia intestinalis*, and *Entamoeba histolytica*, with good selectivity (*SI* > 10). Purification of this fraction allowed the isolation of three SQDGs: the major compound 1-*O*-palmitoyl-2-*O*-myristoyl-3-*O*-(6-sulfo-α-D-quinovopyranosyl)-glycerol, along with small amounts of 1,2-di-*O*-palmitoyl-3-*O*-(6-sulfo-α-D-quinovopyranosyl)-glycerol and a new compound identified as 1-*O*-palmitoyl-2-*O*-oleoyl-3-*O*-(6-sulfo-α-D-quinovopyranosyl)-glycerol.

Plouguerné et al. (2010) isolated MGDGs in a fraction obtained from *Sargassum muticum* collected from the coast of Britanny (France), that inhibited the bacteria *Shewanella putrefaciens* and *Polaribacter irgensii* and the fungi *Halosphaeriopsis mediosetigera*, *Asteromyces cruciatus*, *Lulworthia uniseptata*, *Monodictys pelagica*, all involved in marine microfouling. The inhibitory activity was reported for a concentration of 0.75 mg/l.

The crude ethyl acetate extract of *Fucus evanescens*, collected on the Arctic coast of Ungava Bay, Nunavik (Canada), showed strong antibacterial activity (≥4 log_10_ colony-forming units (cfu) against *Hemophilus influenza*, *Legionella pneumophila*, *Propionibacterium acnes*, and *Streptococcus pyogenes*, when tested at 100 μg/ml. This glycolipid rich extract also inhibited by 3 log_10_ cfu the bacteria *Clostridium difficile* and *Staphylococcus aureus* (Amiguet et al., 2011). Further purification of the glycolipid rich extract led to the isolation and identification of the main compound as the MGDG 2′, 3′-propyl dilinolenate-β-D-galactopyranoside.

El Baz et al. (2013) investigated the structures and biological activities of sulfolipids from the Mediterranean macroalgae *Dilophus fasciola* and *Taonia atomaria*. The authors highlighted antibacterial and antiviral activities from sulfolipids extracts. The major compounds were identified as SQDG and SQMG (sulfoquinovosylmonoacylglyceride).

**Figure 2:**
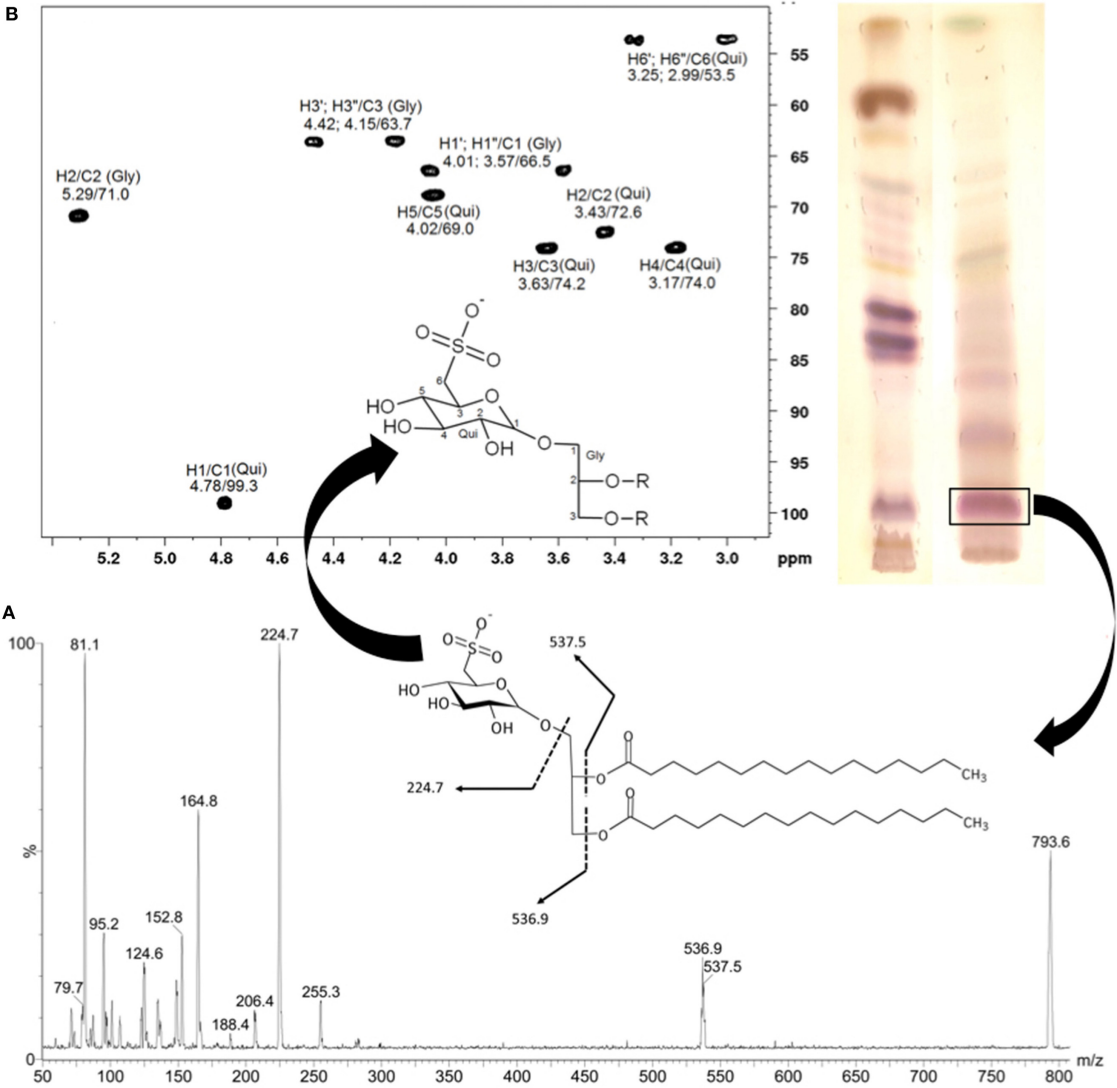
**Electrospray ionization mass spectrum (ESI-MS) of main SQDG from *Sargassum vulgare* (negative mode)**. The fragmentation pathway of the ion at m/z = 794 is compatible with the structure of 1,2-di-*O*-palmitoyl-3-*O*-(6-sulfoquinovopyranosyl)-glycerol **(A)**. The configuration of the anomeric carbon was confirmed analyzing the partial fingerprint spectrum 2D-^1^H/^13^C-HSQC of the polar head group of SQDG **(B)**. Gly, glycerol; Qui, quinovose. (Adapted from Plouguerné et al., 2013).

SGDGs were identified in fractions obtained after the purification of the organic extract of the Brazilian macroalga *Sargassum vulgare*. The main SQDG responsible for the anti-HSV1 and anti-HSV2 activities highlighted was characterize as 1,2-di-*O*-palmitoyl-3-*O*-(6-sulfo-α-D-quinovopyranosyl)-glycerol (Plouguerné et al., 2013). The structure of the SQDG was determined using Electrospray Ionization Mass Spectrometry (ESI-MS) (Figure 2A) and the configuration of the anomeric carbon was confirmed by ^1^H and ^13^C Nuclear Magnetic Resonance (NMR) analysis, based on Heteronuclear Single Quantum Coherence (HSQC) fingerprints (Figure 2B).

Imbs et al. (2013) isolated a highly unsaturated monogalactosyldiacylglycerol (MGDG) from the ethanol extract of *Fucus evanescens*, collected from the west coast of the Iturup Island of the Sea of Okhotsk (Russia). This compound, identified as 1-*O*-(5Z,8Z,11Z,14Z,17Z-eicosapentanoyl)-2-*O*-(6Z,9Z,12Z,15Z-octadecatetraenoyl)-3-*O-β-D-galactopyranosyl-sn-glycerol*, inhibited the growth of human melanoma cells with an IC_50_ = 104 μM.

## Rhodophyta

Ohta et al. (1998) isolated the SQDG KM043 from *Gigartina tenella*, collected at Sagami Bay, Kanagawa, Japan, that demonstrated inhibition of DNA polymerase α, DNA polymerase β and HIV-reverse transcriptase type 1, with respective IC_50_ values of 0.25, 3.6, and 11.2 μM. The structure of the SQDG was identified as 1-(1′-*O*- α-D-sulfoquinovosyl)-2-palmitoyl-3-[5″ (E), 8″ (E), 11″ (E), 14″ (E), 17″ (E)-eicosapentaenyl]-syn-glycerol.

Al-Fadhli et al. (2006) isolated three distinct fractions containing polar glycolipids from the soluble fraction of crude methanolic extract of *Chondria armata*. The structure of the glycolipids was elucidated using multidimensional NMR techniques and ESI-MS in the positive ion mode. The most active fraction showed significant growth inhibition of the bacteria *Klebsiella* sp., the yeast *Candida albicans* and the fungus *Cryptococcus neoformans* when tested at the concentration of 130 μg/disc. The main compound present in the fraction was identified as the MGDG 1-eicosapentanoyl-2-palmitoyl-3-*O*-galactopyranosyl-glycerol.

The MGDG lithonoside isolated from the cytotoxic hexane-soluble extract of the Fijian coralline macroalga *Hydrolithon reinboldii* demonstrated moderate growth inhibitory activity against cancer cell lines with a mean IC_50_ value of 19.8 μM (Jiang et al., 2008).

de Souza et al. (2012) isolated an anti-HSV (herpes simplex virus) glycolipid-enriched fraction from the Brazilian macroalga *Osmundaria obtusiloba*. The major compound of the active fraction was identified as the SQDG 1,2-di-*O*-acyl-3-*O*-(6-deoxy-6-sulfo-α-D-glucopyranosyl)-sn-glycerol.

Tsai and Pan (2012) isolated SQDGs from *Porphyra crispata* collected from northeastern Taiwan that inhibited the growth of human hepatocellular carcinoma cell line (HepG2), with an IC50 of 126 μg/ml.

El Baz et al. (2013) investigated the structures and biological activities of sulfolipids from *Laurencia papillosa* and *Galaxaura cylindrica* collected from the Red Sea. The authors highlighted antibacterial and antiviral activities from sulfolipids extracts. The major compounds were identified as SQDG and SQMG.

Anti-inflammatory activity was highlighted for two SQDGs isolated from *Palmaria palmata* (Banskota et al., 2014). The bioactive compounds were identified as (2S)-1-*O*-eicosapentaenoyl-2-*O*-myristoyl-3-*O*-(6-sulfo-a-D-qu-inovopyranosyl)-glycerol and (2S)-1-*O*-eicosapentaenoyl-2-*O*-palmitoyl-3-*O*-(6-sulfo-α-D-quinovopyranosyl)-glycerol and demonstrated nitric oxide inhibitory activity with IC_50_ values of 36.5 and 11.0 μM, respectively.

## Chlorophyta

Wang et al. (2007) isolated a SQDG from the n-butanol fraction of the invasive *Caulerpa racemosa* collected from the South China Sea. The SQDG compound was characterized using spectroscopic methods as (2S)-1,2-di-*O*-palmitoyl-3-*O*-(6′-sulfo-α-D-quinovopyranosyl) glycerol, and was active against HSV-2, with a 50% inhibitory concentration (IC_50_) of 15.6 mg ml^−1^ against both standard and clinical strains of HSV-2.

MGDGs capsofulvesin A and B, along with the MGMG capsofulvesin C, isolated from *Capsosiphon fulvescens* collected from the southern coastal area of Wando (Korea), demonstrated cholinesterase inhibitory activity (Fang et al., 2012).

Islam et al. (2014) revealed for the first time the aldose reductase inhibitory activity of the capsofulvesin A and capsofulvesin B.

Such results highlighted the potential health benefits of *C. fulvescens* in improving neurotransmission as well as in preventing diabetic complications.

El Baz et al. (2013) investigated the structures and biological activities of sulfolipids from the Mediterranean macroalga *Ulva fasciata*. The authors highlighted antibacterial and antiviral activities from sulfolipids extracts, and the major compounds were identified as SQDG and SQMG.

## Conclusion

Among the three phyla of marine macroalgae, Ochrophyta appears as the main source of recently reported bioactive glycolipids, followed by Rhodophyta and Chlorophyta. Within Ochrophyta, the order Fucales was the most reported, followed by Dictyotales.

Among bioactive glycolipids isolated from marine macroalgae, SQDGs, and MGDGs dominated the reports for the last 15 years. Khotimchenko (2003) studied the distribution of glyceroglycolipids in marine macroalgae and highlighted the predominance of SQDG as a characteristic of brown seaweeds from the order Fucales. It would then be logical to expect such order as a major source of bioactive SQDGs. However, according to the recent literature, bioactive SQDGs were more abundant in Dictyotales.

The most reported biological activities for glycolipids from seaweeds were antibacterial, antitumor, and antiviral activities, enhancing the pharmacological potential of these compounds. Less reported, but of significant interest, is the ecological role that may assume glycolipids biosynthetized by seaweeds. Antifouling, and antiherbivory activities were already reported for glycolipids from *Sargassum muticum* and *Fucus vesiculosus*, respectively (Deal et al., 2003; Plouguerné et al., 2010).

Concerning the mechanism of action of glycolipids, it remains until today relatively obscure. Recent studies about synthesis and structural modification of glycoglycerolipids helped to understand the structure-activity relationship. On general point of view, the bioactivities of glycoglycerolipids are related to the sugar moiety, the position of the glycerol linkage to the sugar, the length and location of the acyl chain, and the anomeric configuration of the sugar (Zhang et al., 2014). Polyunsaturated fatty acids (PUFAs) are biologically active compounds, which are abundant components of macrophytic glycolipids (Khotimchenko, 1993a,b, 2003; Sanina et al., 2000, 2004; Raposo et al., 2013). Therefore, it has been hypothesize that fatty acids may be responsible for biological activities highlighted from macrophytic glycolipids (Tsai and Pan, 2012). As the distribution of PUFAs in glycolipids from seaweeds depends from their taxonomic position, it may then be logical to expect some taxa to be more active than others (Sanina et al., 2004). Among glycolipids, the degree of saturation of fatty acid increased in the lines of MGDG->DGDG->SQDG. Concerning SQDGs, the antiviral activity may be related to the presence of the sulfonate group (Plouguerné et al., 2013). The sulfate moiety along with the nature of the fatty acids of the SQDGs are also important for the inhibition of DNA polymerase α and β (Mizushina et al., 1996, 1997; Hanashima et al., 2000, 2001).

Glycoglycerolipids from seaweeds are compounds with both biotechnological potential and ecological interest. Further studies are needed to extend knowledge concerning the mechanism of action of these molecules as well as their distribution between macroalgal species.

### Conflict of interest statement

The authors declare that the research was conducted in the absence of any commercial or financial relationships that could be construed as a potential conflict of interest.
